# Ferroptosis induction by engineered liposomes for enhanced tumor therapy

**DOI:** 10.3762/bjnano.16.97

**Published:** 2025-08-14

**Authors:** Alireza Ghasempour, Mohammad Amin Tokallou, Mohammad Reza Naderi Allaf, Mohsen Moradi, Hamideh Dehghan, Mahsa Sedighi, Mohammad-Ali Shahbazi, Fahimeh Lavi Arab

**Affiliations:** 1 Student Research Committee, Mashhad University of Medical Sciences, Mashhad, Iran; 2 Immunology Research Center, Mashhad University of Medical Sciences, Mashhad, Iran; 3 Department of Medical Biotechnology and Nanotechnology, Faculty of Medicine, Mashhad University of Medical Sciences, Mashhad, Iran; 4 Department of Pharmaceutics and Nanotechnology, School of Pharmacy, Birjand University of Medical Sciences, Birjand, Iran; 5 Department of Biomaterials and Biomedical Technology, The Personalized Medicine Research Institute (PRECISION), University Medical Center Groningen, University of Groningen, 9713 AV Groningen, The Netherlands; 6 Department of Immunology, Faculty of Medicine, Mashhad University of Medical Sciences, Mashhad, Iran

**Keywords:** cancer, ferroptosis, liposome, nanomedicine, stimuli-responsive

## Abstract

Ferroptosis has shown potential therapeutic effects in tumor therapy as an iron-dependent programmed cell death. The induction of ferroptosis is based on lipid peroxidation, the accumulation of iron and reactive oxygen species, and the depletion of glutathione. Nowadays, various nanoparticles are reported for ferroptosis-based therapy. Among them, engineered liposomes have received more attention due to their biocompatibility, low immunogenicity, and flexibility in chemical and structural modifications. The present review focuses on the mechanisms of ferroptosis and its induction by engineered liposomes to improve tumor therapy. It also highlights the fascinating outcome of liposome-mediated ferroptosis in overcoming the obstacles to cancer therapy, along with the limitations and possible future directions.

## Review

### 1 Introduction

Cancer is defined by irregularities in the processes that control cell division, leading to the survival and rapid spread of cancerous cells. Despite significant advances in medical science and technology, cancer is still a disease with limited therapeutic options. Metastasis and recurrence of cancer lead to high disability and mortality, and the exact mechanisms are still unclear. Current chemotherapy faces challenges, including non-specificity, toxicity to healthy cells, the development of stem-like cells, and the progression of multidrug resistance [1]. Drug resistance is a major obstacle in cancer therapy and is closely linked to alterations in cancer metabolism [2–4]. Changed metabolic pathways allow cancer cells to grow faster than usual, adapt to restricted nutrient conditions, and develop drug resistance [3]. There is still a gap in the efficacy of various cancer therapies despite numerous methods that have been developed to reduce mortality, alleviate chronic pain, and improve quality of life [5]. In 2021, Dixon initially introduced the concept of a new form of cell death called ferroptosis [6]. This form of cell death relies on iron and is not related to apoptosis, distinguished by the accumulation of lipid ROS. Ferroptosis clearly differs from necrosis, apoptosis, and autophagy in terms of cellular morphology and function [7]. Recently, studies have indicated that ferroptosis can be utilized for cancer therapy since it effectively eliminates cancer cells and reverses drug resistance [8–10]. The main advantage of ferroptosis lies in its cell death mechanism, which bypasses multidrug resistance and improves conventional chemotherapy, highlighting its remarkable therapeutic efficacy [8]. Ferroptosis treatment holds potential for addressing a range of conditions, including liver diseases such as non-alcoholic fatty liver disease, cardiovascular diseases like atherosclerosis, neurological disorders, and chronic wound healing [11–13]. Ferroptosis is a unique approach for the simultaneous treatment of cancer cells with chemotherapy, radiotherapy, and immunotherapy to increase the sensitivity of tumor cells to these treatments and improve the efficacy of these treatments [14–16]. New technologies, innovative methods, and interdisciplinary research will drive progress in ferroptosis research and its medical applications [13].

Recently, nanotechnology has expanded its advantages and impact on cancer therapy and diagnosis [17]. Therapies based on nanotechnology, such as the progressive delivery of nanoscale drugs, can provide precisely targeted treatment of malignant tissue with fewer side effects than traditional approaches [5,18]. Key benefits of nanodrug delivery systems include precise targeting, controlled release over time, prolonged half-life, and reduced systemic toxicity [19]. Liposomes, as lipid-based nanoparticles, hold promise for improving cancer therapies as they can encapsulate various anticancer molecules [20]. A liposome typically consists of a hydrophobic phospholipid bilayer and a hydrophilic core. Depending on the physicochemical properties of the drug, this type of structure allows the entrapment of both hydrophilic and hydrophobic drugs [1]. Liposomes, like other nanosystems, have many benefits, such as prolonged systemic blood circulation, reduced drug toxicity, improved pharmacokinetics, the ability to release drugs in a controlled manner, and the ability to target tumors. However, certain limitations also exist, including off-target accumulation and fast clearance [21]. This review explores the design of liposome structures aimed at inducing ferroptosis through various pathways to enhance cancer therapy. We also discuss stimuli-responsive liposomes and evaluate their effects on the immune system.

### 2 Ferroptosis

A recently recognized regulated condition of iron-dependent cell death is called ferroptosis [7,22]. The Latin term “ferrum” (iron) and the Greek term “ptosis” (to fall) are the roots of the name ferroptosis [23]. Ferroptosis is a non-apoptotic cell death, a sub-branch of programmed cell death [24]. This type of cell death was first introduced in 2012 [6]. Ferroptosis differs from known forms such as apoptosis, necrosis, pyroptosis, and autophagy. Like apoptosis, ferroptosis is a mitochondrial-related process in which the mitochondrial membrane is wrinkled and condensed, and its protrusions disappear [25]. In addition to mitochondria, membrane-bound organelles are also involved in ferroptosis. Ferroptosis is triggered by lysosome failure, Golgi stress-associated lipid peroxidation, and oxidative stress associated with the endoplasmic reticulum [23]. Ferroptosis immediately alters the phenotype and function of immune cells [26]. The intricate relationship between lipid metabolism, cysteine, and iron has been recognized as a critical regulator of ferroptosis [23]. The imbalance between oxidative damage and antioxidants is the fundamental biochemical process behind ferroptosis [27]. Common features of ferroptosis include decreased glutathione (GSH) levels, accumulation of ROS in cells, and increased lipid peroxidation, which is a common biomarker of ferroptosis [22]. In general, ferroptosis begins with the Fenton reaction, in which unsaturated lipid molecules are oxidized by •OH radicals produced when iron ions and H_2_O_2_ are combined [28]. An iron-containing porphyrin molecule called hemin can also trigger ferroptosis independently of GSH deficiency (or GSH depletion). Hemin initiates this reaction as an iron catalyst to respond to the excess H_2_O_2_ [29]. Another way of triggering ferroptosis is the oxidation of unsaturated lipids by an iron redox couple, which can also lead to ferroptosis. This process of inducing ferroptosis is especially valuable in situations where Fenton reaction-dependent ferroptosis might be compromised due to elevated levels of GSH and reduced levels of H_2_O_2_, which are commonly observed in tumor cells. Note that iron-induced oxidation of unsaturated fat in the absence of H_2_O_2_ requires the simultaneous presence of Fe^3+^ and Fe^2+^ ions [28]. Induction of ferroptosis could be a valuable strategy to overcome tumor resistance to apoptosis or anticancer drugs and increase the effectiveness of cancer cell killing. It also contributes to the T cells’ immune response against cancer [25]. Ferroptosis and tumor suppressors, such as the p53 molecule, are linked, meaning that ferroptosis can be enhanced or prevented by p53 [30]. Moreover, inflammatory activation are remarkably linked by ferroptosis through some signaling pathways, such as JAK-STAT and NF-κB [27]. Mechanisms involved in ferroptosis are discussed in detail in the next section.

#### 2.1 Mechanisms of ferroptosis

**Figure 1:**
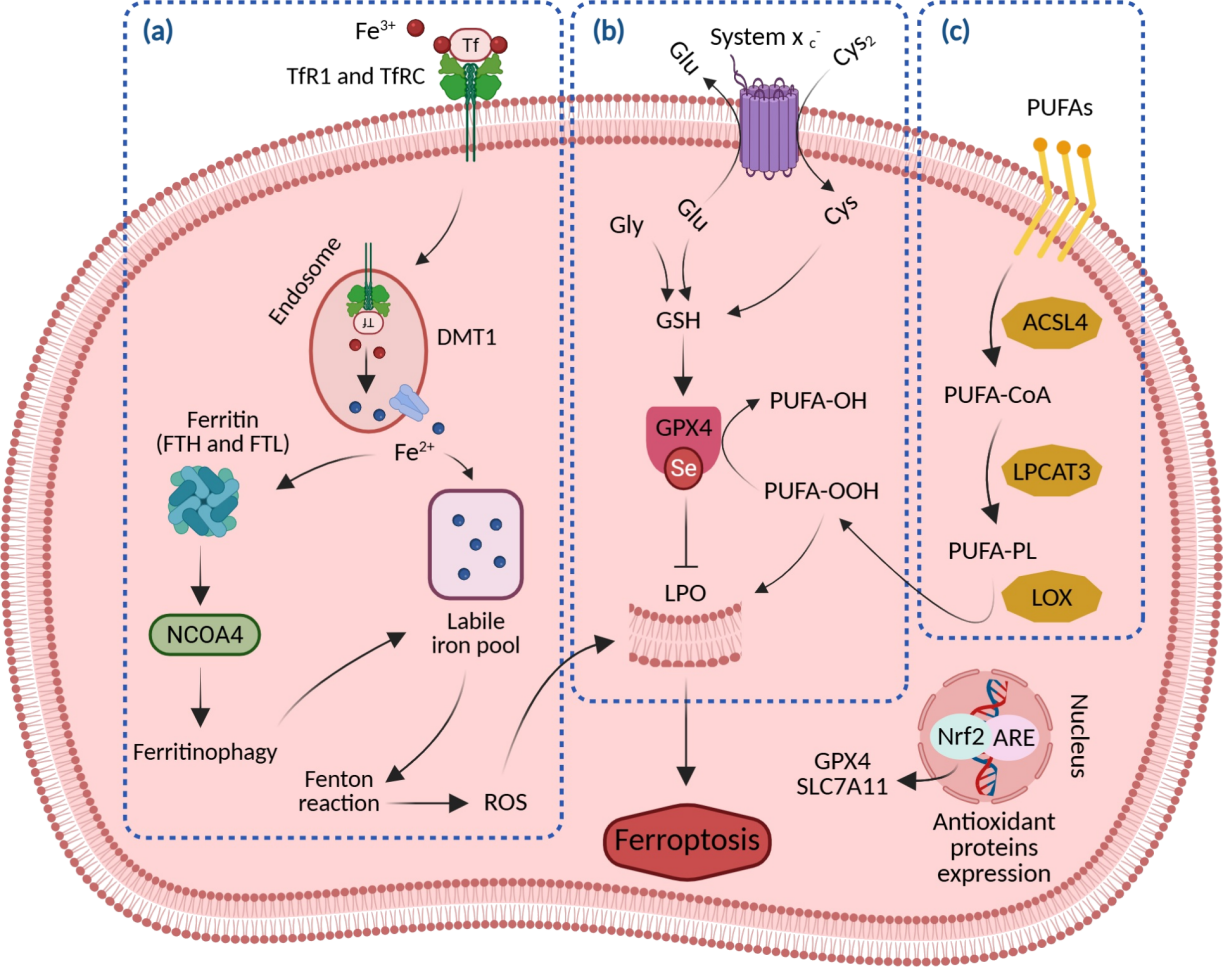
Ferroptosis mechanisms include (a) iron-mediated pathway, (b) amino acid-mediated pathway, and (c) lipid-mediated pathway. In contrast, pathways such as NRF2 can hinder the initiation of ferroptosis by transcriptionally stimulating the expression of SLC7A11 and GPX4, thereby aiding in maintaining cellular balance. Figure 1 was created in BioRender. Shahbazi, M. (2025) https://BioRender.com/3jbuki0. This content is not subject to CC BY 4.0.

Glutathione peroxidase 4 (GPX4) and cystine-glutamate transmembrane (xc^−^) system are considered primary signaling pathways regulating ferroptosis, but iron metabolism and lipid peroxidation are necessary prerequisites for the initiation of ferroptosis. The role of iron metabolism is characterized by the generation of ROS and oxidative stress conditions, while the peroxidation of lipids under these oxidative conditions causes intracellular damage that leads the cell into programmed death by ferroptosis [31–32]. Despite the impressive progress in ferroptosis research, studying its fundamental mechanisms is still ongoing [33]. Here, we investigate the pathways associated with iron, lipids, and amino acids involved in the ferroptosis process, demonstrated schematically in Figure 1.

**2.1.1 Lipid-mediated pathway.** Aside from their multiple functions within the cell, fatty acids also serve as components of phospholipids, which are essential for cell membrane formation [34]. There are three types of fatty acids, namely, polyunsaturated fatty acids, monounsaturated fatty acids, and saturated fatty acids. These fatty acids are converted into phospholipids through the esterification process. When ROS interact with phospholipids containing polyunsaturated fatty acid chains (PUFA-PLs), lipid peroxides are formed, serving as the primary triggers of the ferroptosis process [35–36]. Lipid peroxides cause damage to the bilayer membrane, and structural and functional changes in proteins and nucleotide acids lead to toxic effects in the cell and initiate the ferroptosis cascade [37–38]. Due to the instability of lipid peroxides, it is not easy to detect them. Nevertheless, the secondary products of lipid peroxidation, such as malondialdehyde (MDA) and 4-hydroxynonenal (4HNE), can be used as markers for ferroptosis [39–40]. Considering the role of fatty acids and phospholipids as precursors of lipid peroxide production, enzymes that produce fatty acids and phospholipids may regulate the ferroptosis pathway. For example, acyl-CoA synthetase long-chain family member 4 (ACSL4) and lysophosphatidylcholine acyltransferase 3 (LPCAT3) play a unique role in the initiation of ferroptosis [41]. In addition to the non-enzymatic pathway associated with ROS, lipid peroxidation can also occur via the enzymatic pathway related to lipoxygenase (LOX) enzymes, which cause direct oxidation of PUFA-containing lipids, ultimately leading to the formation of (15-hydroperoxy)-diacylated polyethylene species with two or three oxygen atoms for the onset of ferroptosis [42]. Due to the role of lipid peroxidation in triggering ferroptosis-induced damage, mechanisms have been developed in the cell to neutralize these damaging agents. One of the most important pathways involves the enzyme GPX4. GPX4 has a selenoprotein structure and is controlled by the mevalonate (MVA) pathway [43]. Directly and with the help of GSH, GPX4 causes the consumption of lipid peroxides and the formation of non-toxic lipid alcohol under normal conditions [44]. Changes in GPX4 activity may indicate ferroptosis, which can be monitored by measuring NADPH and phosphatidylcholine hydroperoxide [45–46]. Heat shock proteins can also inhibit ferroptosis by stimulating GPX4 or regulating ROS production, such that HSPB1 and HSPA5 inhibit ferroptosis by inhibiting ROS production and increasing the activity of the enzyme GPX4 [47–48].

**2.1.2 Iron-mediated pathway.** The critical role of iron in ferroptosis was identified through observations of ferroptosis inducers. Researchers found that substances like erastin or RLS3, which disturb the body’s antioxidant system, can cause ferroptosis. However, this process can be stopped by using iron chelators like deferoxamine (DFO). Elevated intracellular iron levels can enhance ROS production during the Fenton reaction [6]. Additionally, iron can increase the activity of the enzyme lipoxygenase, which, as mentioned in the previous section, is associated with ferroptosis [49]. To investigate the role of iron in ferroptosis, we should first familiarize ourselves with its metabolism within the cell. Transferrin is responsible for ferric iron (Fe^+3^) transportation in the body. Due to the role of this transporter in the supply of iron in the cell, it has recently been introduced as one of the specific markers of ferroptosis [50]. Like transferrin, lactoferrin also plays a role in iron transport [51]. Cells absorb iron-bound transferrin with the help of transferrin receptor 1 (TFR1) during receptor-bound endocytosis. In addition to this pathway, iron absorption can also occur with the help of actin filaments, one of the components of the cytoskeleton, via the receptor (TFRC) [36]. The iron required by the cell, in the form of ferrous iron (Fe^+2^), is produced by six transmembrane epithelial antigens of the prostate 3 (STEAP3) in the acidic environment in the endosome. Fe^+2^ is then transported into the cytoplasm by the transporter for divalent metals (DMT1). Iron has various functions in the cell and can be used in different enzymatic processes or stored in ferritin within the cell during a process with the help of the chaperones poly-(rC)-binding protein 1 (PCBP1) and PCBP2 [52–53]. Iron is present in the cytosol in the form of ferritin and pool of accessible iron ions, called labile iron pool (LIP) [54–55]. Ferritin stores excess iron and keeps labile iron in a non-toxic and inorganic state [56]. Iron storage proteins play a unique role in the inhibition of ferroptosis. Inhibition of ferritinophagy by suppressing nuclear receptor coactivator 4 (NCOA4) and preventing ferritin degradation by lysosomes limits the incidence of ferroptosis [57]. In some cases, ferroportin (FPN) can remove iron from the cell. According to the study by Bao et al., loss of FPN leads to iron accumulation and induction of ferroptosis and Alzheimer’s progression in mice [58]. ROS production determines the leading role of iron in ferroptosis during the oxidation reactions of hydrogen peroxides in LIPs under the name of the Fenton reaction [59–60]. In addition, iron regulatory proteins (IRPs) such as IRP1 and IRP2 regulate the expression of DMT1 and TfR1, ferritin, and FPN1 by binding to the mRNA of iron response elements (IREs). Under iron deficiency conditions, IRPs induce the expression of iron-enhancing proteins by binding to IREs. The induction of IRPs is associated with a stimulation of ferroptosis [59,61–62].

**2.1.3 Amino acid-mediated pathway.** Glutamine and the system xc^−^ are effective in producing lipids and the production of the antioxidant GSH [63–64]. The role of GSH as a coenzyme for GPX4 in reducing lipid peroxides is known, with its formation relying on the three amino acids cysteine, glutamic acid, and glycine. The importance of cysteine arises from the fact that studies have shown that cystine (Cys2), the oxidized form of cysteine (Cys), is necessary for the growth of mammalian cells [65]. The need for this amino acid can be prevented by vitamin E with antioxidant function [66]. As a result, the role of cysteine in the regeneration of GSH with antioxidant function has been determined [67]. Cysteine can be produced intracellularly in some cells, such as fibroblasts, macrophages, hepatocytes, and vascular smooth muscle cells, from the amino acid methionine. Still, most cells, including cancer cells, cannot produce endogenous cysteine [68–70]. A range of delivery mechanisms contribute to supplying cells with the cysteine they require, including the system ASC (alanine, serine, cysteine-preferring) and LAT-2 (large amino acid transporter 2) [69]. In neuronal cells, cysteine uptake is supported by EAAC-1, part of the xag^−^ system (a sodium-dependent transporter family) with a strong affinity for acidic amino acids like glutamate and aspartate, while in glial cells xag^−^ system can also transport cysteine [71–72]. The xc^−^ system is a transmembrane transport protein with a high affinity for cysteine, composed of Solute Carrier Family 7 Member 11 (SLC7A11) and Solute Carrier Family 3 Member 2 (SLC3A2) as a heterodimer and plays an essential role in restoring GSH and coping with oxidative conditions by transporting cysteine into the cell. This transporter transfers glutamate to transport cysteine into the cell; therefore, its activity and the system xc^−^ are also inhibited by the increase in extracellular glutamate. For this reason, this transporter is considered a common biomarker for ferroptosis [40,73]. Cancer cells are highly dependent on elevated levels of the iron–sulfur cluster biosynthetic enzyme cysteine desulfurase (NFS1). By blocking NFS1 and inhibiting cysteine transport, ferroptosis was induced in vitro, which reduced tumor growth [74]. The glutamine metabolic pathway, glutaminolysis, is effective in the process of ferroptosis. Studies show that blocking glutaminolysis also inhibits ferroptosis [75].

Several other enzymatic pathways play a role in the regulation of ferroptosis. Coenzyme Q10, which is produced by the MVA pathway, acts as an antioxidant and prevents ferroptosis by suppressing lipid peroxidation. Ferroptosis inhibitor protein 1 (FSP1), previously known as apoptosis-inducing factor mitochondrial-related 2 (AIFM2), has been recognized as an inhibitor of ferroptosis. FSP1 is brought to the plasma membrane by N-terminal myristoylation as an oxidoreductase. Then, it reduces ubiquinone (CoQ10), another byproduct of MVA metabolism, to the lipophilic free radical scavenger panthenol (CoQ10H2). This process helps to prevent the buildup of lipid ROS in the membrane in the absence of GPX4 because FSP1-CoQ is an antioxidant system that acts only on GPX4-depleted cells [76–78]. It is also important to note that its functionality is restricted to the cell membrane [76]. Despite the role of mitochondria in oxidative metabolism and as a site of glutaminolysis, their role in ferroptosis is unclear and controversial. Also, peroxidation of their membrane phospholipid, cardiolipin, has not been observed in ferroptosis. Nevertheless, it may theoretically play a unique role as it contributes to metabolic functions such as the ETC carbon transfer chain. Also, mitochondria can support the process of ferroptosis by dissipating cysteine [36,60,79]. Considering the role of the transcription factor Nuclear Factor Erythroid 2-related Factor 2 (NRF2) in the transcription of genes involved in redox reactions and managing oxidative stress, this factor can be considered an attenuator of ferroptosis [80–81]. NRF2 can impede the initiation of ferroptosis by transcriptionally stimulating the expression of SLC7A11 and GPX4, thus contributing to maintaining cellular balance [82]. p53 has a dual function in stimulating or suppressing ferroptosis; however, in most cases, it stimulates ferroptosis. For example, by inhibiting the expression of SLC7A11, it reduces the uptake of cysteine and sensitizes the cell to ferroptosis [83].

#### 2.2 Targeting ferroptosis in cancer therapy

Some types of cancer are naturally more susceptible to ferroptosis due to distinct metabolic characteristics [84]. PUFA-ePLs produced in peroxisomes serve as substrates for lipid peroxidation. Clear-cell renal cell carcinoma (ccRCC) cells show elevated levels of PUFA-ePLs, likely due to their increased expression of alkylglycerone phosphate synthase (AGPS, an essential enzyme in the synthesis of PUFA-ePLs), which makes them susceptible to ferroptosis [84–85]. In small-cell lung cancer (SCLC), SCLC cells that are non-neuroendocrine exhibit significantly higher sensitivity to ferroptosis compared to neuroendocrine SCLC cells, which can be attributed in part to the overexpression of ether phospholipids (ePLs) synthesis enzymes in non-neuroendocrine SCLC cells and their elevated levels of PUFA-ePLs [86]. Additionally, triple-negative breast cancer cells were observed to be sensitive to ferroptosis. This sensitivity has been attributed to various metabolic characteristics of these cells, such as their high levels of polyunsaturated fatty acids (PUFAs), increased labile iron pool, and weakened GPX4–GSH defense mechanism [35,87]. Additionally, cancer cells that are resistant to therapy and exhibit certain cellular characteristics are susceptible to ferroptosis; for instance, cancer cells displaying a mesenchymal phenotype often accumulate PUFAs due to elevated expression of zinc finger E-box binding homeobox 1 (ZEB1), elongation of very long-chain fatty acid protein 5 (ELOVL5), or fatty acid desaturase 1 (FADS1), which make them vulnerable to ferroptosis. In a similar manner, dedifferentiated melanoma cell subtypes are marked by an accumulation of PUFAs and a lack of reduced GSH, making these cells susceptible to ferroptosis [84].

Due to their vast therapeutic potential, ferroptosis inducers have attracted significant attention in cancer research. Additionally, different nanomaterials have been created to trigger ferroptosis or enhance the effectiveness of ferroptosis inducers locally. Moreover, increasing evidence indicates that ferroptosis contributes, at least in part, to the tumor-suppressive effects of several traditional cancer treatments, such as radiotherapy, chemotherapy, targeted therapy, and immunotherapy, and that ferroptosis inducers could enhance the effectiveness of these treatments by promoting tumor ferroptosis. Hence, inducing ferroptosis through ferroptosis inducers may represent a promising therapeutic approach for targeting cancers with particular characteristics [84].

As small molecules, ferroptosis inducers can trigger ferroptosis through various ways: (I) GSH is the most frequently utilized antioxidant inside the cell and is essential for maintaining antioxidant enzymes like GPX4. The production of GSH relies on the uptake of cysteine mediated by system xc^−^ [88]. The inhibition of system xc^−^ by erastin and its analogs triggers ferroptosis. Glutamate [89], sorafenib [90], and IFNG/IFNγ [91], are some agents that can trigger ferroptosis through system xc^−^. (II) Hindering GPX4 results in the direct buildup of lipid peroxidation. For instance, various compounds with electrophilic chloroacetamides, like RSL3, trigger ferroptosis by forming covalent bonds with and blocking selenocysteine activity at the active zone of GPX4 [46,88]. Also, nitrile oxide electrophiles, specifically ML210, JKE-1674, and JKE-1716, trigger ferroptosis through their interaction with selenocysteine as a binding site [88]. (III) Organic peroxides are chemical compounds with one or more oxygen bonds. The bond between the two oxygen atoms (O–O) can be easily disrupted, leading to the formation of alkoxy radicals (•RO). These organic peroxides are commonly employed to induce oxidative damage in cellular models. Artemisinin derivatives, such as artesunate, belong to the category of organic peroxides that can effectively trigger cell death [88,92–93]. (IV) An accumulation of excess free iron within cells directly triggers lipid peroxidation and then ferroptosis. The degradation of drug-activated ferritin raises the iron levels inside cells, enhancing their susceptibility to ferroptosis. Furthermore, the stimulation of ferritin breakdown through endocytosis elevates the amount of free iron within cells, leading to increased ferroptosis [88,94–95]. Hence, iron overload can be achieved by ferric citrate [96–97] or hemin [98] for this purpose. (V) Other species can also effectively induce ferroptosis in addition to those above. For example, erianin can facilitate ferroptosis by enhancing the levels of calmodulin [99]. Also, hindering FSP1 activity using iFSP1 leads to lipid peroxidation [77].

As discussed before, specific cancer types are susceptible to ferroptosis, and leveraging this susceptibility to trigger ferroptosis offers new possibilities for cancer therapy. Importantly, in various cancers, particularly lung and breast cancer, cancer cells exhibit increased susceptibility to ferroptosis compared to their normal epithelial counterparts in laboratory studies. This information underlines the potential for creating targeted treatment strategies to induce ferroptosis in tumors while minimizing harm to healthy tissues [84,100]. However, different mechanisms that allow cancer cells to evade ferroptosis contribute to their resistance. Disrupting these mechanisms can help re-sensitize ferroptosis-resistant cancer cells or tumors to ferroptosis. Resistance to ferroptosis, which can be influenced by specific oncogenic genes, may be overcome by inhibiting the expression or function of the proteins produced by these genes [84,101–103]. And finally, as we said before, it is becoming more recognized that various standard cancer treatments (such as radiotherapy) can initiate ferroptosis. Thus, enhancing ferroptosis through the use of ferroptosis inducers might enhance their overall effectiveness [84]. For example, in response to radiotherapy, cancer cells develop adaptive reactions, such as upregulating SLC7A11 or GPX4 expression, to fight induced ferroptosis. Accordingly, ferroptosis inducers targeting either SLC7A11 or GPX4, along with radiotherapy, can radiosensitize tumor cells by promoting ferroptosis [104–106].

### 3 Liposomes

Liposomes are spherical vesicles composed of one or more phospholipid bilayers and have been investigated as drug carriers due to their unique properties and versatility [107]. Glycerophospholipids, sphingomyelin, and cholesterol are the main components of liposomes. Glycerophospholipids influence the biophysical properties of liposomes; longer hydrocarbon chains can lead to a denser membrane structure and improve drug retention. Lipid degradation is significantly reduced when liposomes containing sphingomyelin are exposed to acidic conditions. Cholesterol helps organize lipid chains and regulates membrane rigidity and flexibility, which affects several functions, including liposome lifespan and controlled cargo release [108]. Liposomes are highly biocompatible, making them ideal for the targeted delivery of drugs to tumor sites. By encapsulating both hydrophilic and hydrophobic therapeutic agents, they improve the solubility and efficacy of drugs, especially those with poor aqueous solubility. Liposomes reduce the impact on healthy tissue while delivering strong drug concentrations directly to the tumor, minimizing the harmful side effects of chemotherapy [109]. The synthesis and characterization of liposomes are crucial steps to ensure their efficacy and safety as drug delivery systems [110].

#### 3.1 Design and engineering of liposomes

Numerous liposome preparation methods affect their properties, including size, layer structure, and encapsulation efficiency (EE). These techniques can be categorized into conventional approaches such as thin film hydration, reverse phase evaporation, solvent injection, detergent removal, and newer innovations. Also, modern techniques are further developments or adaptations of conventional approaches. These methods allow for better control of liposome size, lamellarity, and drug loading. For example, microfluidics, membrane contactors, and pressure-controlled processes can improve EE, reduce organic solvent residues, and improve scalability and reproducibility [111–113].

The most commonly used synthesis method for clinical liposome formulations is the thin film hydration/Bangham technique [113]. DepoFoam liposome technology utilizes a proprietary liposome formulation that can be administered by injection and provides sustained release of the encapsulated drug over an extended period. DepoFoam liposomes have been used for various therapeutic agents, including antibiotics, analgesics, and anticancer drugs [114]. Microfluidics enables the production of liposomes with precise control over size, composition, and EE. Microfluidics-based methods can produce liposomes with uniform size, which is critical for achieving consistent therapeutic effects [115–116]. Moreover, the membrane contactor method can be scaled up for large-scale production of liposomes. Furthermore, the pressure-controlled method enables the production of liposomes with specific sizes and compositions. This method can produce liposomes with high EE and stability [117]. These new technologies in liposome synthesis offer improved methods for producing liposomes with customized properties, which increased their potential as drug delivery systems for various therapeutic applications [114,117–119].

The optimal method of liposome synthesis for drug delivery depends on various factors, such as the desired properties of the liposomes, the type of drug to be administrated, and the intended application. However, recent advances in liposome synthesis have highlighted the effectiveness of microfluidics as an innovative method that trumps traditional methods, such as thin film hydration, to produce liposomes and lipid nanoparticles [113,120]. Microfluidics offers exceptional control over particle size, lower variability, higher EE, and better scalability, making it the most advanced and practical approach for nanoparticle production [115,119–120].

#### 3.2 Characterization of liposomes

Characterization of liposomes is important to ensure their quality and performance as drug delivery systems. Several techniques are commonly used to analyze the properties of liposomes, including size, lamellarity, surface charge, and EE [110,121]. Dynamic light scattering (DLS) is commonly used to determine liposome size and size distribution. Transmission electron microscopy (TEM) and atomic force microscopy (AFM) can be used to image liposome morphology and determine lamellarity. Zeta potential measurements assess the surface charge of liposomes, which plays a crucial role in determining their stability and interactions with biological systems. EE can be determined by separating the liposomes from the non-encapsulated drug using techniques such as size exclusion chromatography or centrifugation, followed by quantification of the encapsulated drug. Other characterization techniques include nuclear magnetic resonance (NMR) spectroscopy to study membrane fluidity and phase transitions, fluorescence spectroscopy for investigating drug–liposome interactions, and capillary electrophoresis to analyze liposome–drug interactions and drug release [110,121].

Different techniques are used to evaluate the characteristics of liposomes [122–124]. In the case of average particle size, DLS and microscopy techniques such as scanning electron microscopy (SEM), TEM, cryogenic TEM, and AFM are used to determine the size of liposomes [122]. The size of liposomes is a crucial parameter that influences their biodistribution, cellular uptake, and drug release. DLS is a widely used technique to determine the size distribution of liposomes in solution [110,125]. DLS measures the fluctuations in light intensity generated by the Brownian motion of liposome particles and allows for the calculation of their hydrodynamic size [110]. Furthermore, microscopy techniques make the size and morphology of liposomes directly visible [110,125]. In particular, cryo-TEM is advantageous because the samples do not need to be stained or fixed, and the natural structure of the liposomes is preserved [110].

The surface charge of liposomes, often expressed as zeta potential, is an important parameter that influences their stability and interaction with biological systems. Electrophoretic light scattering is often used to measure the zeta potential of liposomes [110,125]. Cryo-TEM and ^31^P NMR are used to evaluate the lamellarity of liposomes, which refers to the number of bilayers of lipids in the vesicles [122]. ^31^P NMR provides information on membrane fluidity and phase transitions, while cryo-TEM allows for direct observation of the number of lipid bilayers [110]. X-ray diffraction, differential scanning calorimetry, and thermogravimetric analysis are used to study the phase behavior of liposomes [122]. These methods play a crucial role in exploring the structural and functional properties of liposomes, which are essential for their successful application in drug delivery systems.

The EE of liposomes, which determines the amount of drug loaded into the vesicles, can be evaluated by various analytical methods. High-performance liquid chromatography (HPLC) and capillary electrophoresis (CE) are widely used methods to quantify the encapsulated drug [110,125]. HPLC separates the liposomes from the free drugs and enables the determination of the EE [110]. CE offers advantages, such as low sample and buffer consumption, short analysis times, and the possibility of investigating interactions between drugs and liposomes directly in the separation capillary [125]. The physicochemical properties of liposomes, including size, surface charge, EE, and stability, directly influence their biological interactions and therapeutic potential. These characteristics are particularly crucial in designing liposomes for ferroptosis induction, as they determine the efficiency of iron delivery, lipid peroxidation facilitation, and drug release kinetics. In the following section, we explore how these engineered liposomes contribute to ferroptosis induction and tumor therapy.

#### 3.3 Applications and advancements in liposomal drug delivery systems

Liposomes are highly promising nanocarriers for drug delivery, owing to their exceptional features such as shielding encapsulated substances from physiological degradation, extending drug half-life, enabling controlled release of drug molecules, and excellent biocompatibility and safety. Beyond medicine, they have also found widespread applications in the food and cosmetic industries. Nevertheless, the full potential of liposomes remains untapped, as only 14 liposomal products are currently available on the market [126].

Among the most important developments is the creation of PEGylated liposomes, in which the surface of the liposome is coated with polyethylene glycol (PEG). PEGylation can significantly improve liposome stability, prolong circulation time and reduce clearance through the reticuloendothelial system [118,127]. PEGylated liposomes, also known as stealth liposomes, have been shown to have a higher drug loading capacity and better therapeutic efficacy [118,128].

**Figure 2:**
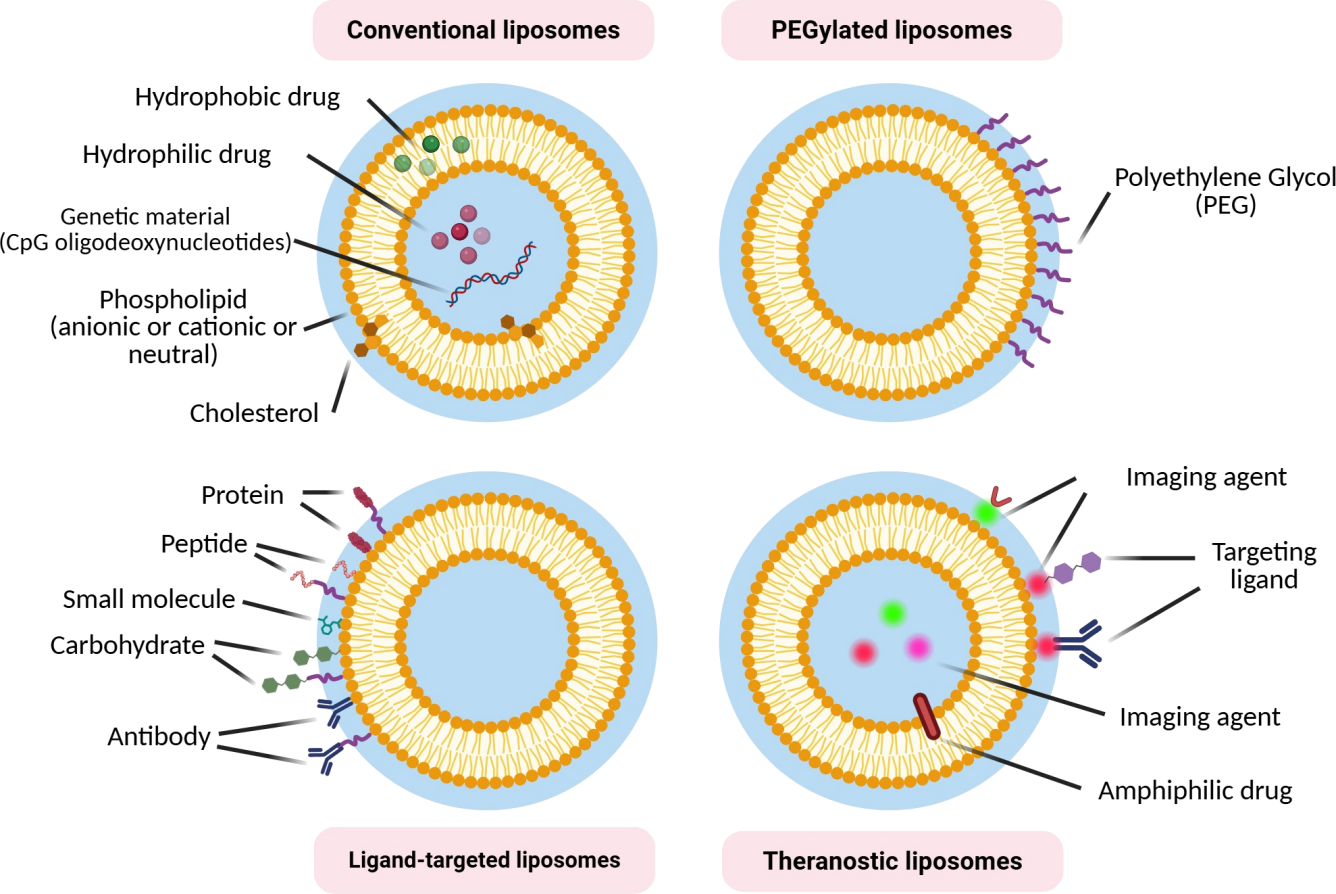
Different types of liposomes are categorized by chemical and structural modifications. Figure 2 was created in BioRender. Shahbazi, M. (2025) https://BioRender.com/yitiaj5. This content is not subject to CC BY 4.0.

Additionally, liposomes can be surface-modified with various ligands such as antibodies, peptides, carbohydrates, and aptamers to achieve targeted drug delivery to specific cells or tissues [118,129]. These ligand-targeted liposomes can bind to receptors on target cells and be internalized by receptor-mediated endocytosis, which improves the accumulation of the drug at the desired site [130–131]. By integrating multiple components, such as targeting ligands, imaging agents, and therapeutic drugs, into a single liposomal formulation, theranostic liposomes can be created that enable simultaneous diagnosis and therapy [132–133]. However, adding multiple components can complicate the manufacturing process and increase costs, requiring careful optimization and scale-up strategies. As shown in Figure 2, there are different types of liposomes. Liposome technology without PEG can sometimes limit the targetability of liposomes [114].

Another innovation is the development of stimuli-responsive liposomes, which release their payload in response to triggers like pH, temperature, or enzymatic activity. Stimuli-responsive technology, based on specific physiological conditions found in tumors compared to healthy tissue, aids in the spatiotemporal release of cargo. For example, pH-sensitive liposomes use the acidic tumor microenvironment to achieve targeted drug release, reducing systemic toxicity and enhancing therapeutic impact [134–135].

According to recent studies, hybrid systems can overcome the disadvantages of each component of the hybrid system. Hydrogel systems included with liposomes demonstrate exceptional chemical tunability, biocompatibility, and biodegradability. These hybrid platforms offer enormous potential for facilitating tissue regeneration or for the localized and controlled delivery of medicine [108]. Liposome–nanoparticle hybrids integrate the benefits of both liposomes and nanoparticles. These hybrids provide a synergistic approach to treating complicated diseases like cancer by enabling multimodal medicines, such as combining chemotherapy with photothermal or photodynamic therapy. These hybrids are especially promising in cancer therapy. For instance, theranostic liposome–nanoparticle hybrids integrate therapeutic agents with imaging capabilities, allowing for simultaneous treatment and real-time tumor response monitoring [136]. Liposome–exosome hybrids are also of great interest, combining the advantages of synthetic liposomes with the natural properties of exosomes to create stable systems that have greater drug loading capacity and targeting specificity [137].

#### 3.3 Challenges and limitations of liposomal drug delivery

Engineered liposomes hold promise for drug delivery and ferroptosis induction, but their potential toxicity and immunogenicity require careful consideration. Although the phospholipid bilayer of liposomes has the advantage of resembling cells, one of the main causes of chemical instability is the possibility of oxidation or hydrolysis processes. After the hydrolysis of free fatty acids, compounds are formed that are toxic to the human body and lower the pH value of the environment. Antioxidants such as flavonoids and vitamins C and E can be included in the formulation of liposomes to stop oxidation [138]. While designed to reduce systemic toxicity, some formulations, particularly cationic liposomes, can be cytotoxic to macrophages and disrupt immunomodulatory signaling. Liposomal immunogenicity depends on factors like surface charge, size, and PEGylation, with positively charged liposomes often triggering stronger immune responses, including complement activation and rapid clearance by the mononuclear phagocyte system. Moreover, PEGylated liposomes may induce anti-PEG antibodies, leading to accelerated blood clearance [139–140]. These challenges highlight the need for precise liposomal design strategies that balance therapeutic efficacy with minimal off-target effects, ensuring safer and more effective clinical translation.

The ability to firmly entrap the medications or genes and stop premature leaking before they reach the target presents another difficulty when using liposomes for drug and gene delivery. Effective lipid binding of medications and genes is necessary to overcome this obstacle [126]. Moreover, the increasing number of physicochemical variables in liposomal formulations makes it difficult to evaluate their pharmacokinetics, pharmacodynamics, and toxicology after administration, which further complicates their clinical translation [141]. The incorporation of surface modifications, coatings, or ligands to improve liposomal functionality can complicate the manufacturing process, increase production costs, and pose a challenge for large-scale production in the context of good manufacturing practice [138].

### 4 Role of liposomes in ferroptosis induction

Given the specific design and properties of the liposomes discussed in the previous section, their role in triggering ferroptosis becomes critical. The ability of liposomes to encapsulate and deliver iron, lipid peroxides, and ferroptosis-inducing agents plays a central role in their therapeutic potential. This section examines the mechanisms through which these liposomes facilitate ferroptosis in tumor cells. As already explained, liposomes are nowadays increasingly preferred because they can transport both hydrophilic and hydrophobic substances and offer additional advantages such as biocompatibility, biodegradability, low toxicity, lack of immunogenicity, and flexibility for chemical and structural modifications [142–143]. Recent developments in liposomes focus on modifying their chemical and structural properties to target cancer cells and trigger offensive responses and mechanisms in the vicinity of tumors [143]. As shown in Table 1 below, most of the mechanisms and markers of ferroptosis in the studies using engineered liposomes are based on increased lipid peroxidation (LPO) accumulation, MDA, decreased GPX4, ROS production, and decreased GSH. In the following, the mechanisms of ferroptosis induced by engineered liposomes are discussed in detail.

Nanotechnology-based drug delivery systems bound to specific ligands have the potential to improve drug delivery efficacy. Most transporters exhibit expression in specific domains and thus provide suitable drug delivery targets to enhance absorption in particular areas [144]. Therefore, engineered liposomes have been developed to deliver targeted ferroptosis agents to tumor cells to improve tumor therapy, which is discussed here. For example, Celastrol (Cel) can inhibit the proliferation of liver tumor cells. Cel can induce ferroptosis and cause cancer cell death through the generation of ROS, reduced GSH levels, and increased Fe^2+/^Fe^3+^ and LPO levels. Cel has also been shown to increase the expression ratio of Bax/Bcl-2 and facilitate cyto-C to trigger the caspase-3-related mitochondrial apoptosis pathway. Cel induces these two types of cell death by targeting the voltage-dependent anion channel 2 (VDAC2) protein [145]. Nevertheless, the side effects of Cel pose a significant obstacle to its potential clinical use [145–146]. Therefore, Luo et al. developed altered Cel-based liposomes (AGCL) using alkyl-*N*-octyl-β-ᴅ-glucopyranoside with the idea that incorporating Cel into alkyl-Glu-modified liposomes could improve its ability to target tumors and reduce potential side effects. The particle size and zeta potential of AGCL were reported to be approximately 83.41 nm and −19.1 mV, respectively. The in vitro results showed that alkyl-Glu can potentially increase the uptake of liposomes by cancer cells. In vivo studies showed that the anti-tumor effect of AGCL on hepatocellular carcinoma (HCC) was more substantial than that of Cel [145]. In another targeted therapy, folate-labeled liposomes were prepared by Gai et al. to co-load metallothionein 1D pseudogene (MT1DP) and erastin (E/M@FA-LPs) to improve the bioavailability and efficacy of the drug/gene mixture. The characterization tests revealed that E/M@FA-LP had a spherical shape, a diameter of about 154 nm and a zeta potential of 24 mV. The EE was also reported to be 48.87. TEM extraction results exhibited that E/M@FA-LPs induced distinct changes related to ferroptosis in mitochondria, including reduced cristae, more undersized mitochondria, and increased membrane compaction in A549 and H1299 cells. In addition, increased ROS and MDA levels and decreased GSH levels were detected in cells treated with E/M@FALPs. The anticancer effect of E/M@FA-LPs in vivo showed increased MiR-365a-3p expression and decreased NRF2 expression, which may have played a role in sensitizing NSCLC cells to erastin-induced ferroptosis [147]. Mitochondria-targeted liposomes have also been studied to enhance mitochondrial-associated ferroptosis in bladder cancer in situ. The synthesized liposome was selectively aggregated in mitochondria and caused mitochondrial lipid peroxidation and ROS by deactivated dihydroorotate dehydrogenase [148].

Targeting lung cancer based on the synergistic effect of ferroptosis and apoptosis is hampered by two obstacles, that is, low intracellular Fenton levels and insufficient aggregation of healing agents in the lung. To overcome these obstacles, a liposome was developed and administrated by inhalation. The diameter of the metal polyphenol network hybrid liposome was 198 nm with a spheroidal shape and a zeta potential of approximately −15 mV. Nebulization resulted in approximately 8.2 times higher lung accumulation than intravenous injection. Co-delivery of a gallic acid-Fe metal polyphenol network and dihydroartemisinin revealed a joint effect on increasing the level of intracellular Fenton-like reaction. Also, decreased expression of GPX4 and increased expression of caspase 3 protein were detected. Moreover, it could simultaneously trigger the accumulation of ROS, LPO, and MDA in the body and increase oxidative stress by further reducing GSH levels. These results indicate a synergistic effect of ferroptosis and apoptosis [149].

**Figure 3:**
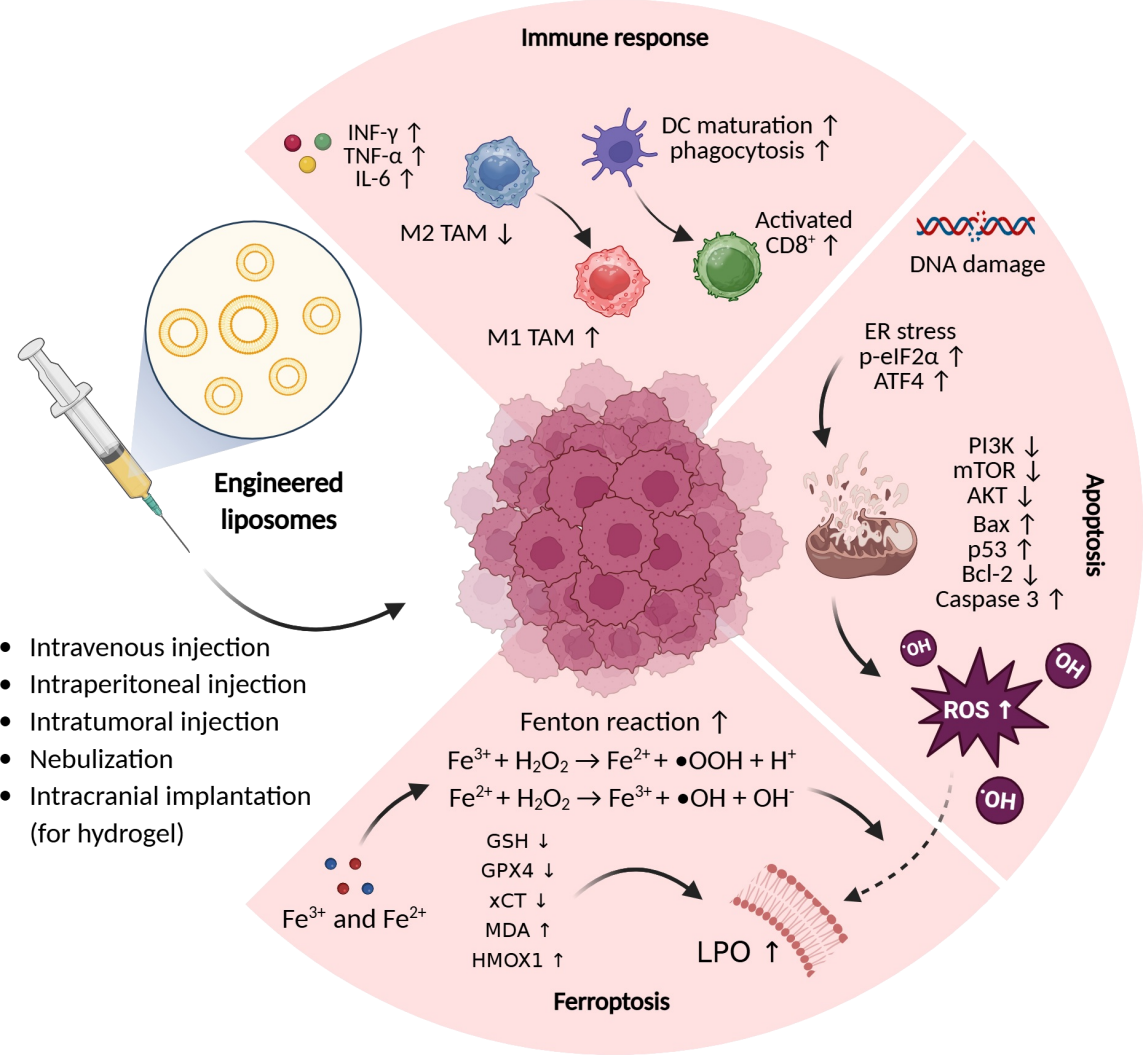
The main mechanisms of enhanced cancer therapy include induction of ferroptosis and apoptosis and immune regulation employing engineered liposomes targeting ferroptosis. Figure 3 was created in BioRender. Shahbazi, M. (2025) https://BioRender.com/3fb01o3. This content is not subject to CC BY 4.0.

In a study, glucose oxidase (GOx) was attached to a manganese sulfide (MnS) shell and encapsulated with a liposome. The hydrodynamic size of the liposomes was about 150 nm. The EE of MnS-GOx nanoparticles in liposomes was 63%. GOx was able to convert glucose into gluconic acid. Gluconic acid induced the degradation of MnS and then enhanced the release of H_2_S and Mn^2+^. In addition, H_2_O_2_ can enhance the Fenton-like reaction triggered by Mn^2+^. The results suggest that the combination of chemodynamic and gas treatment could trigger mitochondrial damage and the formation of mtROS, resulting in the production of LPO active substances and finally inducing ferroptosis [150]. Figure 3 summarizes the key mechanisms of enhanced cancer therapy, which include induction of ferroptosis, apoptosis, and immunoregulation using engineered liposomes targeting ferroptosis.

**Table 1:** A list of studies focusing on targeted ferroptosis by engineered liposomes.^a^

Effector loaded reagents	Main component of liposome	Size (nm)/EE (%)	In vivo study model	Ferroptosis mechanisms	Year	Ref.

ferric ammonium citrate	PC	≈94.6^b^/98.5	4T1/ BALB/c mice	* increase of LPO * reduce intracellular GSH content and GPX4 expression level	2020	[151]
metallothionein 1D pseudogene, erastin	ʟ-α-PC, CHO, DSPE-PEG	≈174^b^/48.87	A549/ BALB/c nude mice	* increase ROS generation * increase MDA * decline GSH levels * intracellular ferrous iron was upregulated	2020	[147]
doxorubicin, sorafenib	DOPE, CHO	≈93^b^/80, 90	4T1/ BALB/c mice	* decrease the intracellular GSH * ROS production * reduction in GPX4 protein level * increase MDA level	2020	[152]
trisulfide bond-bridged DOX homodimeric prodrug (DSSSD), ferric ammonium citrate	PC, DSPE-PEG2k, CHO	≈107.86^b^/87	B16-F10 melanoma/ C57BL/6 mice	* increase of ROS levels * GSH depletion * increase of LPO	2021	[28]
CuCP	DPPC, DSPE-PEG-2000- Maleimide, CHO	≈107^b^/ 80	CT26/ Balb/c nude	* raise the production of •OH * gathering of LPO * quicken GSH depletion * repress the GPX4 expression * raise the levels of MDA and 4-HNE	2022	[153]
gallic acid-ferrous iron	DPPC, CHO, DSPE-mPEG 2K	≈200^b^/55	MCF-7/ADR/ Balb/c nude mice	* elevate intracellular Fe(II) accumulation * induce LPO * deplete GSH * inactive GPX4 * enhance Fenton reaction	2022	[25]
copper peroxide nanodots, artemisinin	DPPC, CHO mPEG2k-DSPE	≈100^c^/–	LLC/ Balb/c nude mise	* decrease the expression levels of GPX4 and xCT * downregulation of the expression levels of FTH1 * production of ROS	2022	[154]
chlorogenic acid, mitoxantrone, Fe^3+^	HSPC, CHO, PEG-zobenzene-oleinic acid	≈112^b^/–	CT26/ Balb/c mice	* enhance ROS generation * reduce the expression of GPX4 and FACL4	2022	[155]
ferrocene	lecithin, DSPE-PEG, CHO	≈30^c^/43.4	4T1/ BALB/c mice	* induce robust ROS generation * increase MDA * LPO accumulation * decline GSH content * downregulation of the activity and expression of GPX4	2023	[156]
ʟ-buthionine-sulfoximine, hemoglobin, hematoporphyrin monomethyl ether	lecithin, CHO, DSPE-mPEG2000	≈168^b^/89	Conlon 26/ Balb/c nude mice	* decrease of GSH * promote the production of ROS * increase of HMOX1	2023	[157]
dihydroartemisinin	lecithin, CHO, Ca^2+^	≈146^c^/–	A549-luc/ BALB/c nude mice	* ROS accumulation * LPO accumulation * downregulation of the expression of GPX4 * increase MDA	2023	[158]
moexitecan, superparamagnetic iron oxide nanoparticles	DPPC, DSPC, DSPE-PEG2000, CHO	≈143^b^/76.86	HT-29/ BALB/c mice	* reduce the concentration of GSH * increase in intracellular ROS levels	2023	[159]
sorafenib, hemin	DPPC, DSPE-PEG2000, amphiphilic dendrimers	≈100^c^/–	SMMC7721/ BALB/c nude mice	* increased intracellular Fe^2+^ and •OH * robust increase in ROS concentration * GSH depletion * elevated MDA	2023	[29]
ferric ammonium citrate, sorafenib	PC, mPEG2K-DSPE, CHO	≈99^b^/95.31 and 91.71	4T1/ BALB/c mice	* augment cytoplasmic ROS levels * accumulation of LPO through a decrease in intracellular Gpx4	2023	[160]
brequinar	SPC, DSPE-mPEG2K, CHO, TPP	–	MB49/ C57BL/6 mice	* raise LPO, MDA, and PTGS2 * reduction of the ratio of GSH to GSSG	2023	[148]
ferric ammonium citrate and cisplatin	DSPC, CHO, DSPE-PEG2k, DSPE-PEG2K-FA	≈100^b^/–	4T1/ BALB/c mice	* large production of ·OH * induce a devastating accumulation of LPO * amplify the oxidative stress via additional ROS production	2023	[161]
erastin, temozolomide	hydrogenated SPC, CHO, cRGD-coated DSPE-PEG	≈165^b^/36.88 and 90.4	GL261TR/U251TR-luciferase/ C57BL/6 J and NU/NU mice	* reduce the protein levels of GPX4, xCT and ferritin * reduce the cellular GSH levels * increase the MDA levels * MGMT protein expression inhibition and the p53 expression enhancement * occur the accumulation of oxidative stress	2023	[22]
celastrol	SPC, CHO, DSPE-mPEG2K, *N*-octyl-β-ᴅ-gluco- pyranoside	–	SK-Hep1/ BALB/c nude mice	* induce the generation of ROS * decrease GSH levels * enhance the levels of Fe^2+^/Fe^3+^ and LPO	2023	[145]
gallic acid-Fe^2+^	lecithin and 143B cell membranes	≈100^b^/–	143B/ BALB/c nude mice	* elevate the expression of PTGS2 and LPO * upregulate the expression of NRF2 * ROS generation	2023	[142]
manganese sulfide-glucose oxidase	DSPC, DSPE-PEG-2K, and CHO	≈150^b^/63	PC-3/ nude mice	* reduce GSH * decrease GPX4 activity * LPO accumulation * •OH generation	2024	[150]
FeCl_2_	glucose monohydrate, urea, DSPE-mPEG2k	≈5.6^c^/–	A549/ BALB/c nude mice	* reduce of GSH * increase in the intracellular ROS level * downregulation of intracellular GPX4 expression	2024	[162]
CpG oligodeoxynucleotides	DSPE-PEG2000-fucose, 1,2-dioleoyl-3-trimethyl- ammoniumpropane/DOTAP, ROS-responsive lipid, CHO	–	4T1/ BALB/c mice	* ROS production * induce LPO * increase the MDA * GSH depletion	2024	[163]
dihydroartemisinin	CHO, DSPE-poly(2-ethyl 2-oxazoline)2K, lecithin, FeCl_3_, gallic acid	≈198^b^/98.73	A549-Luc/ BALB/c nude mice	* downregulation of GPX4 * increase Fe^2+^ levels * GSH depletion * induce the accumulation of ROS, LPO, and MDA	2024	[149]
sulfasalazine	lecithin, CHO, PEG2K-DSPE, and folate-PEG2K-DSPE	≈110^b^/67.83	B16F10 tumor-bearing C57BL/6JNifdc mice	* downregulate of GPX4 and FTH1 * upregulate of PTGS2 and ACSL4 * increase ROS levels * increase of MDA * decrease of GSH	2024	[164]
[Ir (PPY^−^)_2_(BAPIP)](PF_6_), [Ir(PIQ^−^)_2_(BAPIP)](PF_6_), [Ir(BZQ^−^)_2_(BAPIP)](PF_6_)	lecithin, CHO, DSPE-mPEG2K	–/87.6, 88.3, and 85.4	A549/ BALB/c mice	* reduction in GSH concentrations * downregulation of GPX4 * increase of HMGB1, and LPO	2024	[165]
[Ir(ppy)_2_(PIP)](PF_6_), [Ir(ppy)_2_(NPIP)](PF_6_), [Ir(ppy)_2_(NNIP)](PF_6_)	HSPC, CHO, 5-cholesten-3b-ol 3,6- dioxovinyloctanedioate and a targeted ASGP-r receptor glycoligand molecule	–/86.54, 89.49, and 89.67	HepG2/ BALB/c nude mice	* downregulation of GPX4 * downregulation of the expression of ferritin * increase in HMGB1 content * decrease GSH content * decrease the ratio of GSH/GSSG * enhance MDA levels	2024	[166]

^a^LPO: lipid peroxidation, GSH: glutathione, GSSG: glutathione disulfide, GPX4: glutathione peroxidase 4, ROS: reactive oxygens species, MDA: malondialdehyde, HMGB1: high mobility group protein 1, PTGS2: prostaglandin-endoperoxide synthase 2, ACSL4: long-chain fatty acid CoA ligase 4, FTH1: ferritin heavy chain 1, NRF2: nuclear factor erythroid 2-related factor 2, xCT: cysteine-glutamate transporter, MGMT: *O*-6-methylguanine-DNA methyltransferase, HMOX1: heme oxygenase-1, FACL4: fatty acid-CoA ligase 4, 4-HNE: 4-hydroxynonenal, PC: phosphatidylcholine, COL: cholesterol, DSPE: 1,2-distearoyl-*sn*-glycero-3-phosphorylethanolamine, PEG: polyethylene glycol, DOPE: dioleoylphosphatidylethanolamine, DPPC: dipalmitoylphosphatidylcholine, mPEG: methoxy polyethylene glycol, DSPC: 1,2-distearoyl-*sn*-glycero-3-phosphocholine, SPC: soybean phosphatidylcholine, TPP: triphenylphosphonium, FA: folic acid; ^b^indicates hydrodynamic diameter; ^c^indicates diameter determined with TEM.

While engineered liposomes facilitate ferroptosis induction, their therapeutic efficacy can be significantly enhanced through stimuli-responsive strategies and combination therapies. Stimuli such as pH, temperature, light, ultrasound, and ROS can trigger controlled drug release, improving selectivity and minimizing off-target effects. Moreover, combining ferroptosis with other therapeutic modalities, such as chemotherapy or immunotherapy, may amplify its antitumor effects. The following section explores these advanced strategies to further optimize ferroptosis-based tumor therapy.

### 5 Stimuli-responsive strategies and combination therapy

**Figure 4:**
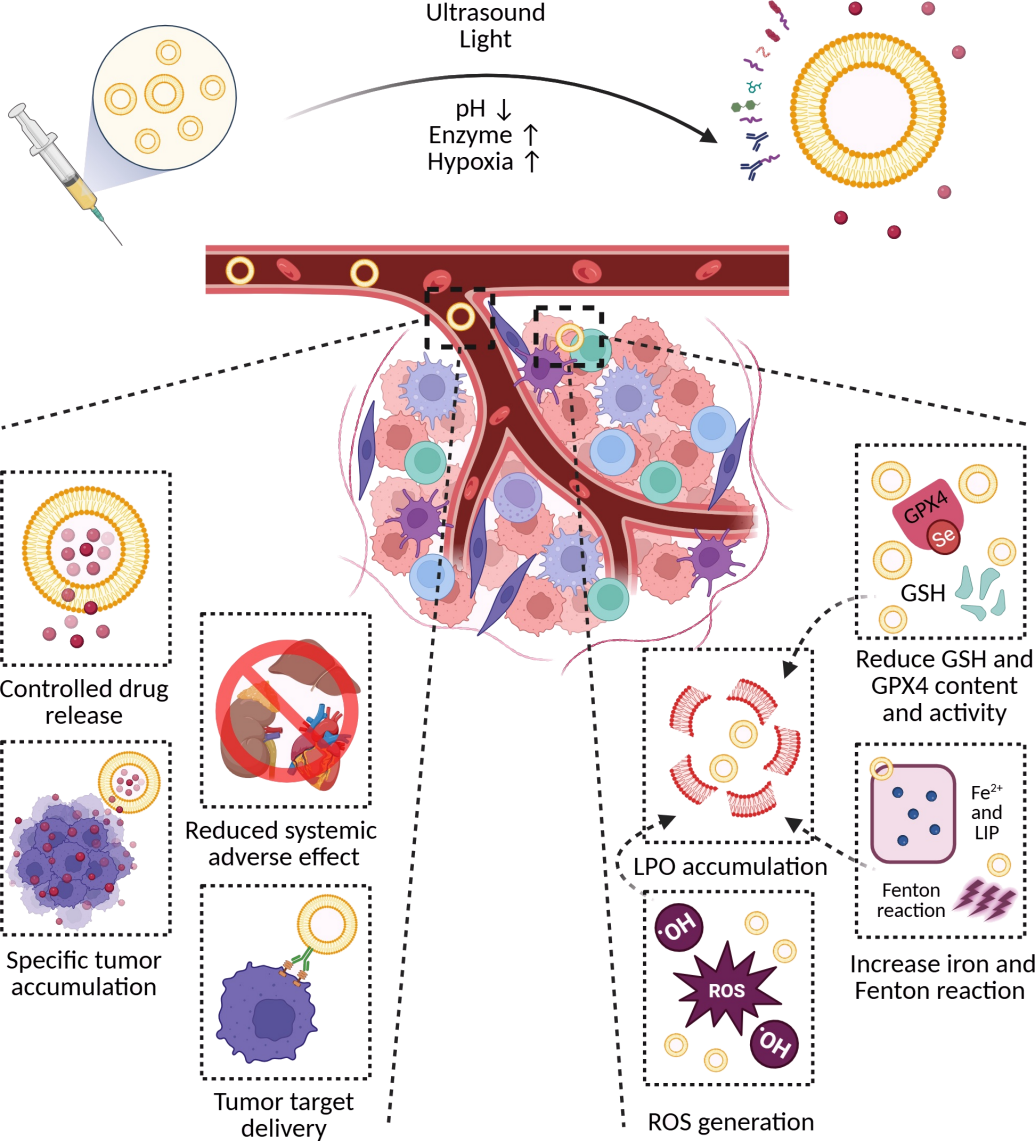
Schematic illustration of ferroptosis-related mechanisms, various types of stimuli-responsive strategies and their main advantages in anti-tumor drug delivery and ferroptosis induction. Figure 4 was created in BioRender. Shahbazi, M. (2025) https://BioRender.com/dgnqa6j. This content is not subject to CC BY 4.0.

Building upon the role of liposomes in ferroptosis induction, the integration of stimuli-responsive release mechanisms and combination therapies offers a promising avenue to enhance therapeutic efficacy. This section discusses various external and internal stimuli that can be leveraged for precise drug release, as well as synergistic therapeutic approaches that potentiate ferroptosis-driven tumor cell death. Recently, stimuli-responsive strategies for nanodrug delivery systems have gained attention due to their potential ability to regulate drug release according to biological and environmental triggers [167–170]. Compared to normal tissue, the tumor microenvironment (TME) typically exhibits increased acidity, ROS and GSH levels, hypoxia, expression of enzymes, and ATP levels because of tumors’ rapid growth and metabolism. Therefore, these stimuli in the TME can be employed as TME-responsive nanomedicine strategies to enhance the efficacy of cancer immunotherapy and reduce side effects through specific aggregation in tumors and regulated release of drugs [171]. In addition to internal stimuli, external stimuli (e.g., temperature, light, or ultrasound (US)) [172] can also be used. The release of drugs from liposomes stimulated by external stimuli offers greater precision in terms of timing and location of release as well as better regulation of drug delivery and dosage [172–173]. Figure 4 shows various stimuli-responsive strategies for inducing ferroptosis to improve cancer therapy using engineered liposomes.

For example, ferrocene-loaded PEG-liposomes were synthesized in spherical form with a diameter of about 30 nm. The measured zeta potential was about −37 mV. The EE of liposomes with ferrocene (a green Fenton catalyst) was calculated to be about 43.4%. Liposome-PEG loaded with ferrocene showed increased ferrocene release in the presence of H_2_O_2_ and under acidic conditions while preventing significant leakage of ferrocene during circulation of body fluids. It has been suggested that acidic tumor conditions favor ferrocene oxidation and the Fenton reaction between ferrocene and H_2_O_2_, resulting in more ferrocene ions and higher LPO levels that disrupt the integrity of the lipid membrane of the liposome, leading to increased ferrocene release. This positive feedback seems to be able to initiate a robust cascade production of •OH and LPOs within the TME [156]. Another pH-responsive liposome also showed better catalytic activity at weakly acidic conditions by combining amphiphilic dendrimers with liposomes. The combination of amphiphilic dendrimers with liposomes significantly enhanced the release of sorafenib at pH 5.5, achieving 83% within 24 h. These pH-responsive liposomes showed controlled release of sorafenib and hemin under TME conditions, enabling ferroptosis–apoptosis therapy of HCC [29].

Malignant tumors require an abundant supply of nutrients to support the rapid growth of cancer cells. Therefore, specific transporters are often overexpressed to meet these nutrient requirements (such as the amino acid transporter B^0,+^ (ATB^0,+^), and the cystine/glutamate transporter (xCT)) [152,174]. Also, matrix metalloproteinase 2 (MMP2) is an enzyme located outside cells that contributes to the development, expansion, and metastasis of tumors, which are more prevalent in various types of cancer [175]. Recently, this enzyme has been used as a stimulus for the release of tumor-specific ligands or drugs [152,176]. Based on this, an MMP2-activated and ATB^0,+^-targeted liposome with doxorubicin and sorafenib (DS@MA-LS) was developed to treat 4T1 tumor cells. The EE of doxorubicin (DOX) in liposomes about 80%, whereas the EE of sorafenib (SRF) in liposomes was approximately 90%. DS@MA-LS showed extensive presence in the bloodstream, selectively aggregated in the tumor area, and cleaved the PEG cover by MMP2-mediated cutting. Then, the linked lysine was exposed, leading to endocytosis of the liposome into the cancer cells by ATB^0,+^. Subsequently, DOX increased ROS levels, and SRF blocked xCT to suppress GSH. As a result, combined stimulation of apoptosis and ferroptosis improved tumor therapy [152].

TME significantly induces hypoxia, which promotes cancer progression. Therefore, hypoxia is another suitable stimulus for controlled drug release in TME and prevention of non-tumor release [155,170]. For example, Chen et al. developed a hypoxia-responsive liposome to coat the nanoelicitors of chlorogenic acid, mitoxantrone, and Fe^3+^ ions, exhibiting a spherical morphology with a hydrodynamic diameter of about 112.73 nm. The drug release tests demonstrated burst cargo release under hypoxic conditions; for instance, over 76.3% of mitoxantrone was released after 10 h of incubation in a hypoxic environment. The in vivo results of this hypoxia-responsive liposome showed remarkably reduced GPX4 and FACL4 expression [155].

Ultrasound technology has found wide application in medicine for diagnostic and therapeutic purposes, primarily because it is affordable, safe, non-ionizing, non-invasive, and easy to use. The energy generated by US can serve as an external stimulus to initiate the release of drugs from nanoparticles [177–178]. US-triggered release can occur through the thermal action of US and the mechanical action of cavitation or radiation forces [177]. US-triggered drug delivery aims to selectively increase the accumulation at the intended target zone [179]. Sonodynamic therapy (SDT) has shown better tissue penetration compared to conventional photodynamic treatment, making it an effective approach for cancer treatment. However, as SDT relies on increased oxygen levels to produce ROS, rapid oxygen depletion may exacerbate tumor hypoxia, compromising its effectiveness as a treatment modality. For example, Yuan et al. prepared liposomes by loading hemoglobin (Hb), ʟ-buthionine-sulfoximine, and hematoporphyrin monomethyl ether (HMME) together. The average hydrodynamic size of loaded liposomes was about 168 nm, and the zeta potential was negative −36 mV. Loaded liposomes significantly triggered early apoptosis at 16.3% and late apoptosis at 38.9%. However, the combination of loaded liposomes and US markedly enhanced early apoptosis to 29.6% and late apoptosis to 56.2%. In contrast, US alone did not result in notable cellular apoptosis. This study showed that liposomes plus US increased ROS, MDA, and 4-HNE levels. In addition, morphological changes of mitochondria associated with ferroptosis were observed in colon 26 cells, which included damaged outer mitochondrial membrane and decreased mitochondrial volume (liposomes plus US group). The in vivo experiments demonstrated possible tumor accumulation after intravenous injection. The Hb contained in the liposomes can supply oxygen to the hypoxic area, which increases the oxygen supply to the tumor and enhances the efficiency of SD. All these results showed that liposomes incorporated with US led to tremendous apoptosis and ferroptosis tumor cell death and modulated TME [157]. In another study, the US-augmented ferroptosis approach was shown to promote phagocytosis of nanoliposome cells, enhance the Fenton reaction, and regulate drug release. Gallic acid-iron (GA-Fe(II)) and DOX-loaded liposomes were prepared with an average diameter of 200 nm, and the EE of Fe and DOX in nanoliposomes was 55.3 and 74.3%, respectively. Furthermore, liposomes loaded with GA-Fe(II) and DOX could induce ferroptosis by promoting intracellular Fe(II) accumulation, generating LPO, decreasing GSH, and inactivating GPX4. Also, the mixture of US irradiation and •OH generation by GA-Fe(II) stimulated the decrease in the expression of PGC-1α and Bcl-2. Finally, drug resistance was abolished, and DOX-induced cell apoptosis was increased [25]. US irradiation also induced drug release and improved the effectiveness of the catalytic response, resulting in the highest healing efficacy in a study by Li et al. Using liposomes and US yielded increased ROS levels and robust mitochondrial damage [154].

Recently, several types of photoresponsive systems have been developed that release drugs on demand in response to illumination with a specific wavelength. These systems are characterized by their non-invasiveness and the possibility of spatiotemporal remote control [178]. It has been documented that ultraviolet (UV) radiation can enhance the Fenton reaction by stimulating the conversion of Fe^3+^ to Fe^2+^ ions through UV-mediated processes [161,180–181]. However, the application of this short-wavelength light for ferroptosis-based cancer therapy is limited due to the limited tissue penetration and rapid attenuation of UV light [161,182]. To overcome this obstacle, upconversion nanoparticles (UCNPs) are considered a promising option for using near-infrared (NIR) light to convert it into visible/UV light without significantly damaging normal tissues and biological samples [161,183].

Beyond enhancing ferroptosis efficiency, stimuli-responsive strategies and combination therapies may also shape the TME and influence immune responses. Ferroptotic cell death is known to release immunogenic signals, potentially triggering immune activation or suppression, depending on the context. The following section explores how ferroptosis induction by liposomes affects the immune system, with implications for immunotherapy and tumor progression.

### 6 Effects on the immune system

Tumors have immunogenic properties similar to other pathogenic entities while also exhibiting unique biological responses. Antitumor immunity involves multiple immune cell types, with the initial step typically involving the presentation of tumor-associated antigens (TAAs) to antigen-presenting cells (APCs), especially dendritic cells (DCs) and macrophages [184]. In association with human leukocyte antigen (HLA) class I and II molecules, TAAs are presented by DCs to CD8^+^ T (CTLs) cells and CD4^+^ helper T cells (Th1 and Th2), respectively. Once activated, Th1 and Th2 subtype cells are also capable of further stimulating CTLs [185]. Programmed cell death 1 (PD-1) and cytotoxic T lymphocyte antigen 4 (CTLA-4) are inhibitory receptors found on T cells that serve to diminish immune responses mediated by these T cells; however, cancer cells take advantage of these inhibitory molecules to promote tolerance to tumors and also lead to T cell exhaustion. Consequently, immune checkpoint inhibitors like anti-CTLA-4, anti-PD-1, and anti-PD-L1 can bind to these inhibitory receptors, effectively restoring the immune response against cancer cells, making them suitable therapeutic targets [186]. Ferroptosis-targeted liposomes can influence these processes to enhance their anti-tumor properties.

To attenuate the inhibitory effect of GPX4 on ferroptosis in the treatment of colorectal cancer, Chen et al. developed a hypoxia-responsive nanoelicitor (HRNE) that effectively exerts ferroptosis by inducing tumor-killing solid immunity. Infiltrating immune cells and the release of cytokines in tumor tissue were studied after treatment with different formulations to investigate the cellular and molecular changes in immune cell subsets. Chlorogenic acid (CA) and mitoxantrone (MIT), two polyphenols suitable for the immune system, self-assembled with Fe^3+^ ions to form self-deliverable nanoelicitors, which were subsequently enveloped by a hypoxia-responsive hybrid liposomal membrane. In the TME, the proportion of IFNγ^+^ CD8^+^ and IFNγ^+^ CD4^+^ T cells was significantly higher in the combination therapy with HRNE than in the control groups. Next, they examined the expression of the xc system by identifying its two main components, that is, the catalytic subunit of SLC7A11 and the regulatory subunit of SLC3A2. This was done because IFN-γ can inhibit the downstream pathway associated with GPX4 and consequently cause ferroptosis. The results revealed that the expression of SLC7A11 and SLC3A2 was significantly lower in tumor tissues with higher IFN-γ expression in the HRNE group. In addition to IFN-γ, the HRNE-treated group showed significantly higher expression of granzyme B, indicating a highly effective activation of the immune system. Flow cytometry was also used after each treatment to examine the phenotypes of TAM. After treatment with CA-based formulations, the M2 TAM population decreased significantly, and the M1 TAM population increased, further reducing the immunosuppressive effect of TME [155].

Huang et al. developed liposome-encapsulated iridium(III) complexes that target mitochondria and cause ferroptosis-induced cell death. This study investigated pyroptosis as a form of cell death different from ferroptosis. In the liposome groups, the results showed morphological features of pyroptotic cells, including cellular expansion and observable balloon-like protrusions along the cell membrane. A lactate dehydrogenase (LDH) release assay was performed, which is considered a reliable indicator of pyroptosis. In this experiment, the release of LDH from the cells indicates a damaged cell membrane, which increases the amount of LDH. The results showed increased LDH content in the groups treated with different liposomes. Additionally, the upregulation of NF-κB, downregulation of caspase 1, and conversion of GSDMD-F to GSDMD-N demonstrate that the generated liposomes can induce pyroptosis [165].

Yuan et al. used an HMME-based liposomal nanosystem (HB liposomes) and developed a US-augmented technique to synergistically enhance the induction of ferroptosis/apoptosis/immunogenic cell death and initiate the reprogramming of TME. After different treatments, they examined the immune cells in the tumor tissue. Treatment with HB liposomes with the US (25%) compared to HB liposomes alone (21.2%), the US alone (14.2%), and blank (11.7%) significantly increased the number of mature dendritic cells (mDCs) (CD11c^+^CD80^+^) in tumor tissue. This indicated that their administration could lead to enhanced DC maturation and T cell activation in the TME as well as the release or exposure of additional damage-associated molecular patterns (DAMPs) such as calreticulin (CRT) or adenosine triphosphate (ATP). Therefore, flow cytometry was used to further investigate the infiltrating T cells. The results showed that more CD8^+^ cytotoxic T cells (32.9%) were found in tumors that received the double treatment compared to tumors treated with HB liposomes only (24.3%), US only (12.3%) or blank (10.8%). However, no significant differences were found in other immune cells, such as CD4^+^ T cells, macrophages, and NK cells. The anticancer effect of the PD-1 antibody was then investigated in combination with HB/US liposomes. Notably, the triple treatment (HB liposomes/US plus PD-1 antibody) suppressed tumor growth more effectively than the double treatment. Furthermore, triple therapy with HB liposomes/US plus PD-1 significantly prolonged the life of tumor-bearing mice, although the double treatment prolonged the life of mice [157].

To enhance ferroptosis in bladder cancer cells by targeting their mitochondria in situ, Ding et al. developed a brequinar (BQR)-loaded liposome (BQR@MLipo). They discovered that BQR@MLipo could cause significant ROS generation and mitochondrial lipid peroxidation, leading to ferroptosis of bladder cancer cells. This process also increased the release of intracellular DAMPs, including CRT, ATP, high mobility group box 1 (HMGB1), and mitochondrial nucleic acid (mtDNA), which could act as an “eat me” signal to promote phagocytosis of apoptotic tumor cells by dendritic cells. Reportedly, the enzyme cyclic GMP-AMP synthase (cGAS) would recognize mtDNA evasion from injured mitochondria, produce the second messenger cyclic dinucleotide of cyclic GAMP (cGAMP), and activate the stimulator of interferon genes (STING). According to the authors’ qPCR study and the Western blot, BQR@MLipo could indeed cause a mtDNA escape. This would then activate the cGAS-STING signaling cascade and cause the secretion of IFN-β, possibly activating APCs. Additionally, MB49 cells exposed to BQR@MLipo showed a considerable increase in P-STING and phosphorylation interferon regulatory factor (P-IRF3) protein levels, suggesting that BQR@MLipo can efficiently induce the STING signaling pathway. After incubation of MB49 cells pretreated with BQR@MLipo with Raw264.7 macrophages, the results showed that MB49 cells exhibited increased susceptibility to phagocytosis by Raw264.7 macrophages after pretreatment with BQR@MLipo. Furthermore, as expected, BQR@MLipo caused a significantly higher IFN-β level. Next, they investigated whether enhanced ferroptosis associated with mitochondria could provide new insights into the malignant progression of antitumor agents and improve the efficacy of immune checkpoint blockade (ICB) on MB49 tumor cells. Treatment with BQR@MLipo+αPD-1 resulted in the broadest zone of necrosis and nuclear diffusion within the tumor tissue. At the same time, the precise staining images and measurement of Ki67 and TUNEL showed that BQR@MLipo+αPD-1 significantly reduced cell division and enhanced apoptosis. The results also showed that BQR@MLipo+aPD-1 stimulated the maturation of 40.9% of DCs (MHCII^+^/CD11c^+^ gate in CD45^+^ cells) in bladder tumor tissue, in contrast to the PD-1 (20.6%) and BQR (21.2%) groups. Moreover, BQR@MLipo+aPD-1 caused the strongest infiltration of CD8^+^ T cells and increased IFN-γ levels. Additionally, BQR@MLipo, in combination with checkpoint blockade immunotherapy, was shown to efficiently inhibit tumor growth without causing harmful side effects [148].

**Figure 5:**
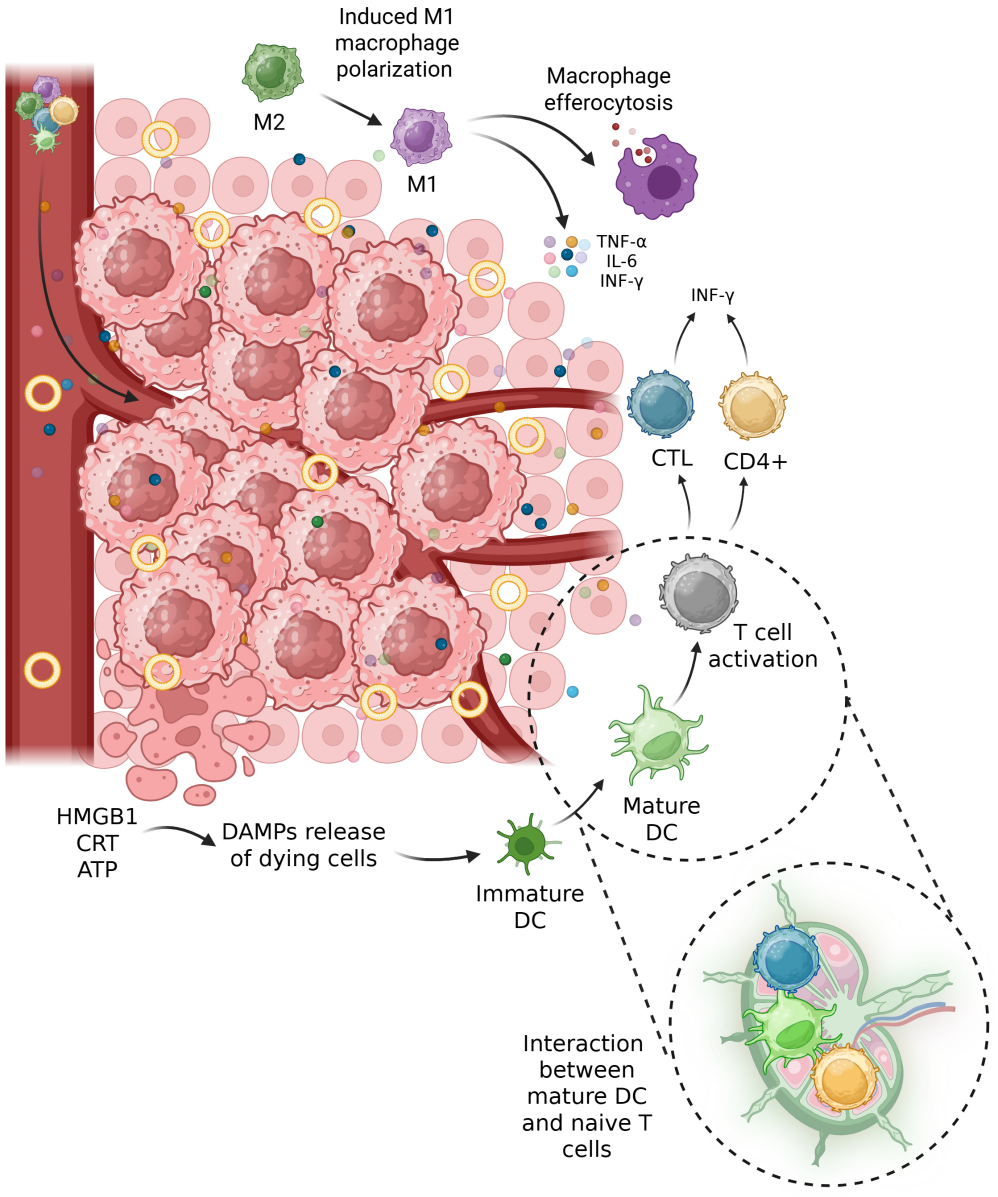
Effects of engineered liposomes on immune system mechanisms to induce an anti-tumor microenvironment. Figure 5 was created in BioRender. Shahbazi, M. (2025) https://BioRender.com/egi47z7. This content is not subject to CC BY 4.0.

For the administration of an immunostimulant, CpG oligodeoxynucleotides (ODNs), and a ferroptosis inducer, arachidonic acid-containing lipids (DAPC), Gao et al. created a targeted fucose-engineered liposome (DAPC-Fuc/CpG). Due to the negative charge, the DCs absorbed the free CpG ODNs very poorly, as evidenced by the observation that the degree of DC maturation after treatment was low. The most effective sample for inducing DC maturation was the targeted liposome. They postulated that the immunostimulatory activity of CpG ODNs in DCs depends on the ability of the liposome and fucose ligand to help DCs internalize the ODNs. In addition, the DAPC-Fuc/CpG combination may facilitate cargo release from liposomes in response to ROS. Cytokines with pro-inflammatory functions, such as interleukin-6 (IL-6) and tumor necrosis factor-alpha (TNF-α), would be released from the mDCs. After treatment with triggered liposomes, imDCs demonstrated significant secretion of TNF-α and IL-6, indicating their maturation. DAPC-Fuc/CpG was more effective than DAPC/CpG in stimulating cytokine release. An in vivo study revealed that all variants of CpG-loaded liposome treatment could inhibit the expression of SLC7A11. DAPCFuc/CpG exhibited the highest SLC7A11 inhibitory property and correlated strongly with IFN-γ concentration between these different compositions. The increased TNF-α and IL-6 concentrations in serum indicate that mDCs release proinflammatory cytokines. After DAPC-Fuc/CpG therapy, the CD80^+^/CD86^+^ subpopulation was greatest in the spleen, indicating a high degree of DC maturation. Additionally, maturation of DCs would stimulate cytotoxic CD8^+^ T cells. Sliced tumor tissue was stained with DAPC-Fuc/CpG to show that the tumor site had the highest concentration of CD8^+^ T lymphocytes. Through the production of IFN-γ and suppression of SLC7A11, activated CD8^+^ T lymphocytes would enhance LPO, especially in the ferroptosis of cancer cells [163]. The effects of engineered liposomes in changing TME to an antitumor microenvironment are illustrated in Figure 5.

### 7 Future perspectives

Although research on ferroptosis-based cancer treatments is rapidly advancing, several challenges still need to be addressed, presenting significant opportunities as well. Since there are already FDA-approved liposome-based drugs [187–189], these engineered liposomes can be taken into the clinical trial phase if appropriate efficacy and safety studies are conducted. A disconnect exists between the advanced pre-clinical research of liposome-based ferroptosis cancer therapy and its transition to clinical application. It is essential to validate innovative liposome-based ferroptosis cancer therapy for enhanced cancer treatment through comprehensive studies of ferroptosis regulatory molecules and signaling pathways. Continuous evaluation of engineered liposome safety and the development of image-guided localized strategies for ferroptosis nanomedicine are necessary for their clinical implementation. Moreover, focusing on economic feasibility, regulatory aspects, safety evaluations, alignment with clinical needs that are currently unmet, and public perceptions will be vital for the successful translation of promising engineered liposome-based ferroptosis cancer therapy. It is also proposed to study the effect of other stimuli, such as temperature, because investigating these models of stimuli-responsive therapy could enhance the circulation half-life and boost accumulation at the tumor site, which is currently minimal due to swift renal clearance. As mentioned above, some of these engineered liposomes had a good effect in promoting the immune response and creating an anti-tumor microenvironment. However, the number of studies conducted in this regard appears to be limited, and it is suggested that future studies focus more on the effects of these structures in promoting the antitumor response. In addition to the quantity, it is also noticeable that the subclasses of T helper and T regulatory cells, which have different functions, were not evaluated. NK cells, which play an essential role in antitumor immunity, were also not investigated. Also, tumor cells typically metastasize locally via the lymphatic system before spreading through the blood, as high GSH and oleic acid levels, along with reduced free iron and ferroptosis, are present in the lymph. Developing lymph node-targeted engineered liposomes could more effectively induce ferroptosis in highly metastatic tumor cells. Hence, understanding the immunogenicity of ferroptosis could offer new insights for designing ferroptosis-based therapies, highlighting the need for further research in this area. Also, the evaluation of signal pathways related to the activation, inhibition, or polarization of different types of these cells can become attractive topics for future studies. In addition, ferroptosis, a crucial mechanism of regulated cell death maintaining organismal balance, has a complex influence on human health. The development of certain diseases is believed to be linked with ferroptosis. Hence, it is vital to enhance the specificity and manage the dosage of ferroptosis inducers to minimize adverse effects on healthy tissues. Furthermore, the variability and adaptability of cancer cells lead to differences in their sensitivity to ferroptosis. Therefore, it is of paramount clinical importance to manipulate the key factors that influence the ferroptosis sensitivity of cancer cells and to prevent malignancies from evading anticancer treatments.

## Conclusion

As mechanisms of ferroptosis have shown potential implications for the treatment of cancer, the use of engineered liposomes to induce ferroptosis has recently been considered. These engineered liposomes displayed promising outcomes in the delivery of ferroptosis inducers. The application of these agents significantly enhanced the induction of ferroptosis in cancer cells; however, the mechanisms involved and their clinical relevance still need further exploration. The favorable properties, such as high loading capacity, low immunogenicity, and straightforward synthesis, powerfully illustrate the potential of these modified liposomes for inducing ferroptosis in the reviewed studies. Additionally, the use of stimuli-responsive versions of these liposomes demonstrated reduced systemic toxicity and increased tumor accumulation. Also, these structures demonstrated promising ability in modifying the tumor microenvironment and were capable of exerting their antitumor effects through immunological alterations, including an increase in M1 macrophages and CTL populations and triggering ferroptosis. Overall, ferroptosis induction through engineered liposomes demonstrated potential anticancer properties. However, more examination is needed to clarify the actual position of this therapy in clinical investigation.

## Data Availability

Data sharing is not applicable as no new data was generated or analyzed in this study.
